# Commentary: A network theory of mental disorders

**DOI:** 10.3389/fpsyg.2017.01305

**Published:** 2017-08-02

**Authors:** Payton J. Jones, Alexandre Heeren, Richard J. McNally

**Affiliations:** ^1^Department of Psychology, Harvard University Cambridge, MA, United States; ^2^Institute of Psychological Sciences, Université Catholique de Louvain Louvain-la-Neuve, Belgium; ^3^Institute of Neuroscience, Université Catholique de Louvain Brussels, Belgium

**Keywords:** network theory, network approach, psychopathology, mental disorders, symptoms, etiology, cognitive therapy

Whether mental disorders differ by kind or degree has been a longstanding debate among clinical theorists who favor either a categorical or dimensional approach to psychopathology (McNally, 2011). Yet proponents of both views agree that symptoms reflect a latent entity (e.g., “major depression”) that causes symptom emergence and covariance (Figure 1). Unfortunately, these latent models have serious theoretical and psychometric limitations (Borsboom, 2008).

**Figure 1:**
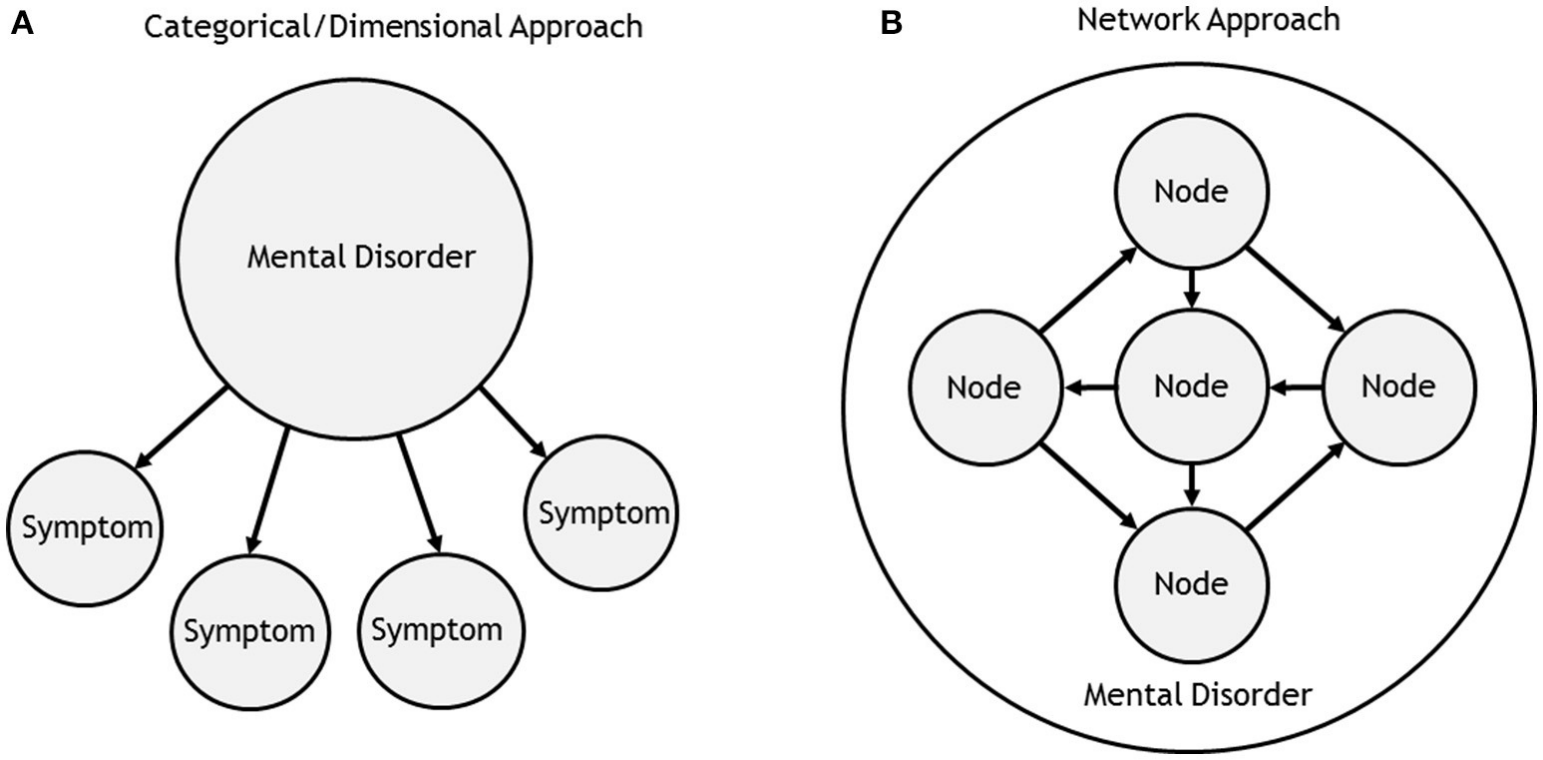
Latent vs. network approach to psychopathology. **(A)** Both Categorical and dimensional approaches to psychopathology assume that a latent entity is causally responsible for symptoms. **(B)** The network approach to psychopathology posits that mental disorders can be explained by the interactions between nodes in a complex network. The relationship between nodes and disorders is mereological (i.e., parts to whole) rather than causal. In the traditional network approach, nodes correspond directly to the “symptoms” in the categorical/dimensional approach. In the expanded network approach, nodes are not limited to symptoms: they may also consist of biological, cognitive, or other individual-level processes.

One alternative to these latent models is the burgeoning “network approach to psychopathology” (Cramer et al., 2010; Borsboom and Cramer, 2013; for reviews, see McNally, 2016; Fried et al., 2017). This new approach conceptualizes a mental disorder as emerging from causal interactions among symptoms, not as an underlying disease entity. Hence, a disorder constitutes a presumably causal network of symptoms (“nodes”) and the connections among them (“edges”).

Taking stock of this growing literature, Borsboom (2017) elucidated four axiomatic principles that characterize the network theory of psychopathology. Principle 1 affirms that mental disorders are best construed as networks emerging from interactions among components (e.g., thoughts, behaviors). Principle 2 holds that components constitutive of networks (“nodes”) correspond to symptoms that appear in diagnostic manuals. Principle 3 states that the structure of psychopathology networks emerges from direct causal connections between nodes. Principle 4 asserts the distinctive phenomenology of diverse mental disorders arises from topological differences in the causal connections among nodes.

Principle 2 indicates that the causal networks responsible for psychopathology consist of *symptoms* that appear in diagnostic manuals. Indeed, almost all extant network studies in psychopathology have been based on symptoms alone (Fried et al., 2017). However, many non-symptoms likely play a causal role in mental disorders. Borsboom (2017) asserts that this is not problematic because all non-symptoms can be described as (1) existing in the “external field” (e.g., a stressor that activates symptoms, but plays no further causal role), (2) constituting a symptom (e.g., neural correlates may constitute a parallel measure of a symptom), or (3) constituting a single symptom-symptom relationship (e.g., describe how one symptom causes another symptom).

Yet not all non-symptom influences on psychopathology can be described in these terms. Non-symptoms can have direct, reciprocal relationships with multiple symptoms (Heeren and McNally, 2016a). Thus, the purpose of our commentary is to suggest expanding Principle 2 of the network theory to include nodes that do not correspond to symptoms.

Consider an important cognitive process in panic disorder: catastrophic misinterpretation of bodily sensations (Clark, 1986). Catastrophic misinterpretation is causally important in the etiology (and the treatment) of panic, and mediates the causal pathway between symptoms (e.g., between accelerated heart rate and fear of dying). Not only does it explain the relationship between symptoms; it can also be caused by symptoms (e.g., having a panic attack may increase catastrophic misinterpretation in the future).

In a network study, Heeren and McNally (2016a) found that the orienting component of attention—a non-symptom node measured in the laboratory—strongly predicted fear of social situations, which predicted avoidance of these situations in people with social anxiety disorder. Social avoidance, in turn, predicted heightened alertness—also measured by a laboratory task—which predicted orienting and fear. This study illustrates how including non-symptom nodes, such as cognitive variables measured in the laboratory, can enrich symptom networks. Other illustrations can be found in recent publications (e.g., Hoorelbeke et al., 2016; Isvoranu et al., 2017).

Rather than being confined to symptoms, nodes in psychopathology networks should consist of variables that (1) vary at the individual level and (2) are plausible causal candidates in the etiology or maintenance of disorders.

By “individual level,” we refer to scale. For example, gender operates on a higher, between-individual level (e.g., gender varies in the population, not in the individual). Such variables reflective of individual differences between persons should not be assumed to exist as causal variables within individuals (Borsboom and Dolan, 2006). On the other hand, neurotransmitters operate on a lower level than the individual (e.g., vary across brain regions). Examples of nodes that vary at the individual level include cognitive processes, beliefs, behaviors, schemas, psychophysiological measures, and symptoms of mental disorders. Hybrid network models (Fried and Cramer, in press) and external moderator models (e.g., de Beurs, 2017) show promise as a means of including components in causal systems that do *not* vary at the individual level.

There are a large number of possible nodes that fit this definition; thus, we suggest that network theorists start by including nodes that are hypothesized to play a *causal* role in psychological disorders according to prominent models of psychopathology (e.g., Heeren and McNally, 2016b). If networks represent causal systems (Principle 3; Borsboom, 2017), it follows that nodes must have causal importance. The requirement of causal importance also renders networks as falsifiable hypotheses: if nodes are not causally important, they should be removed.

Accordingly, we suggest expanding Borsboom's Principle 2 beyond symptoms to include cognitive, biological, and social variables that have seldom been examined in network models. Among others, some examples include: self-beliefs and metacognitive beliefs, information-processing bias for threat-related material, social behaviors, daily-life functional, and occupational impairments, and biological measurements.

Some may have concerns that moving beyond symptoms will “open the floodgates” to overly diverse, confusing networks that undermine parsimonious modeling. Yet this concern is not a persuasive reason for excluding plausible causal elements from networks; striking a balance between precision and parsimony is desirable. Initial studies following this expanded view (Heeren and McNally, 2016a; Hoorelbeke et al., 2016; Isvoranu et al., 2017) have shown that including non-symptoms as nodes in psychopathology networks is both empirically feasible and theoretically enriching. Nevertheless, we suggest caution in selecting appropriate nodes. Adding or removing nodes should be argued on a case-by-case basis and should be accompanied by empirical support that the node in question plays an autonomous causal role in the relevant network.

In conclusion, we propose an expansion of the network theory of psychopathology in which nodes consist of individual-level causal variables. Expanding the network approach beyond symptoms will further strengthen this potentially revolutionary framework for studying psychopathology.

## Author contributions

All authors listed have made a substantial, direct, and intellectual contribution to the work, and approved it for publication.

### Conflict of interest statement

The authors declare that the research was conducted in the absence of any commercial or financial relationships that could be construed as a potential conflict of interest.
